# Direct Evidence of Plasticity within Human Primary Motor and Somatosensory Cortices of Patients with Glioblastoma

**DOI:** 10.1155/2020/8893708

**Published:** 2020-09-22

**Authors:** William R. Gibb, Nathan W. Kong, Matthew C. Tate

**Affiliations:** ^1^Feinberg School of Medicine, Northwestern University, Chicago IL, USA; ^2^Department of Neurological Surgery, Northwestern University Feinberg School of Medicine, Chicago IL, USA; ^3^Department of Neurology, Northwestern University Feinberg School of Medicine, Chicago IL, USA

## Abstract

Glioblastoma multiforme (GBM) is a devastating disease without cure. It is also the most common primary brain tumor in adults. Although aggressive surgical resection is standard of care, these operations are limited by tumor infiltration of critical cortical and subcortical regions. A better understanding of how the brain can recover and reorganize function in response to GBM would provide valuable clinical data. This ability, termed neuroplasticity, is not well understood in the adult human brain. A better understanding of neuroplasticity in GBM could allow for improved extent of resection, even in areas classically thought to have critical, static function. The best evidence to date has demonstrated neuroplasticity only in slower growing tumors or through indirect measures such as functional MRI or transcranial magnetic stimulation. In this novel study, we utilize a unique experimental paradigm to show direct evidence of plasticity via serial direct electrocortical stimulation (DES) within primary motor (M1) and somatosensory (S1) cortices in GBM patients. Six patients with glioblastoma multiforme in or near the primary motor or somatosensory cortex were included in this retrospective observational study. These patients had two awake craniotomies with DES to map cortical motor and sensory sites in M1 and S1. Five of six patients exhibited at least one site of neuroplasticity within M1 or S1. Out of the 51 total sites stimulated, 32 (62.7%) demonstrated plasticity. Of these sites, 14 (43.7%) were in M1 and 18 (56.3%) were in S1. These data suggest that even in patients with GBM in or near primary brain regions, significant functional reorganization is possible. This is a new finding which may lead to a better understanding of the fundamental factors promoting or inhibiting plasticity. Further exploration may aid in treatment of patients with brain tumors and other neurologic disorders.

## 1. Introduction

Gliomas are the most common primary malignant brain tumors with 17,000 new diagnoses per year [1]. Glioblastoma multiforme (GBM), which is the most common type of glioma, also carries the highest mortality. One key treatment that may increase survival in GBM patients is gross-total resection [2]. Gross-total resection can be limited by the desire to spare functional areas which have been infiltrated by tumor. This practice comes from the fact that there is no direct evidence to date of neuroplasticity in GBM, meaning the primary cortices have not been shown to reorganize in response to GBM. These critical areas have been presumed to have a static function that, once resected, will leave the patient with critical neurologic impairment. The aim of this study is to examine neuroplasticity in GBM, using serial direct electrical stimulation (DES) of primary motor (M1) and sensorimotor (S1) cortices in patients harboring GBM to better understand if plasticity can occur in these regions that are thought to be static.

A number of studies have suggested that the brain is capable of remodeling itself in the setting of glioma [3–14]. However, many of these studies used indirect measures of brain function, mainly through imaging modalities such as functional magnetic resonance imaging, positron emission tomography, and transcranial magnetic stimulation. There are few studies that use DES [12, 15–18], a more direct indication of function, and even fewer that look longitudinally in order to capture plasticity over time [12, 16]. Further, studies conducted using DES have included patients with low-grade gliomas (LGG), given the longer survival time and fewer overt neurologic deficits compared to GBM, the most common type of high-grade glioma (HGG). GBM patients, in comparison to LGG patients, are more likely to have overt neurologic deficits with more rapid onset. Thus, we hypothesized that there would be a greater likelihood of capturing cortical reshaping in progress by examining GBM patients as opposed to after it has already occurred. Our objectives were to examine this plasticity over time in the most aggressive primary brain tumor and to gain insight on possible mechanisms of plasticity. These insights into the plastic potential of the brain may lead to advances in management of brain tumor patients such as more aggressive tumor resections, resulting in decreased mortality and morbidity, and more intelligent timing of repeat surgery. GBM have an abysmally low 5-year survival rate of 4.7%, thus making advances in this field a top concern [1]. The present study is the first to present longitudinal evidence of primary motor and sensory cortex plasticity from patients with GBM who have undergone repeat direct electrical stimulation, the “gold standard” for mapping cortical function in humans.

## 2. Materials and Methods

### 2.1. Patient Selection

This was a retrospective observational study design. Patients were considered for the study if they were adults (>18 years old) who underwent two serial awake craniotomies with DES mapping of primary motor (precentral gyrus) and sensory (postcentral gyrus) cortex as part of their usual care for GBM from 2013 to 2016. Of the 207 patients that underwent awake surgery for resection of GBM, a total of 6 patients were identified that fit these criteria. All surgeries and brain mapping were performed by a single neurosurgeon (MCT) at Northwestern Memorial Hospital. Prior to each surgery, objective testing of motor and sensory functions was performed by a neurologist or neurosurgeon as part of standard care. Data from these exams were extracted from the electronic medical record. A patient was defined as having a neurologic motor deficit if they scored less than 5/5 on confrontational strength testing, had a primary complaint of weakness in their face, arms, or legs, or had a pronator drift. Likewise, a neurologic sensory deficit was defined by the clinical exam as well. The institutional review board at Northwestern University reviewed and approved this study, and consent was obtained according to the Declaration of Helsinki.

### 2.2. Direct Electrical Stimulation-Based Intraoperative Brain Mapping

In all procedures, a sedation-awake-sedation technique was employed. Briefly, remifentanil 0.1 *μ*g/kg/min was titrated for respiratory rate of 8-12/min and supplemented with propofol 10-25 *μ*g/kg/min for sedation. Selective scalp blocks were performed using a mixture of tetracaine 1% (60 mg) and lidocaine 1% with epinephrine 1:200,000 (30 ml). Six scalp nerve blocks are performed on each side: supratrochlear (1 ml), supraorbital (1 ml), zygomaticotemporal (5 ml on the side of surgery, 3 ml on the contralateral side), auriculotemporal (3 ml), lesser occipital (3 ml), and the greater occipital (3 ml).

Next, the craniotomy was performed. Following craniotomy, all sedation was held and the dura opened sharply. Upon exposure of the cortical surface and prior to tumor resection, direct cortical mapping was performed. The methods for DES have been described previously [19, 20]. Stimulation of the brain surface was performed using an Ojemann handheld cortical bipolar stimulator with ball-tip electrodes spaced 5 mm apart (60 Hz, biphasic, 1 msec pulse duration, 2-3 sec stimulation duration). Brain stimulation occurred at 1 cm spacing for the entire exposed cortical surface. Mapping was first performed starting at 1 mA and increased up to a maximum of 8 mA or until motor and/or sensory changes were seen. Once the threshold intensity was found, the remainder of the mapping session occurred at that same intensity. A positive sensory site was noted if the patient expressed dysesthesias in the face, arms, or legs during stimulation. A positive motor site was noted if the patient had involuntary movement of the face, arms, or legs. Positive stimulation sites were marked with a paper label while negative sites remained unmarked. After mapping was completed, the exposed cortical region and corresponding map was photographed digitally for additional analysis.

### 2.3. Grid-Based Analysis

**Figure 1:**
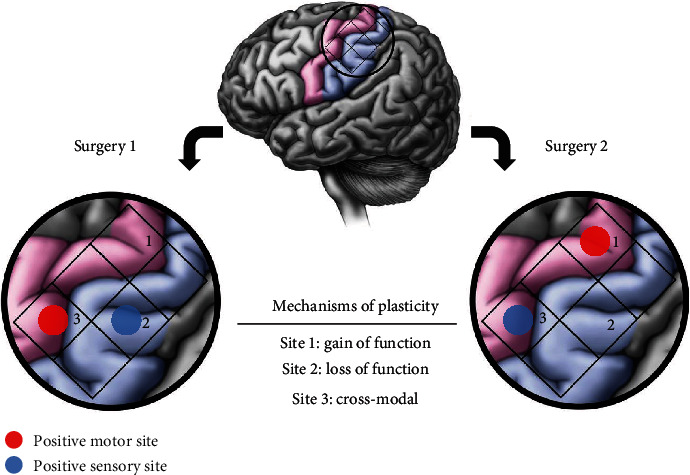
Mechanisms of plasticity. Plasticity occurred in three presumed mechanisms which are illustrated in this figure. Gain of function sites were negative to stimulation at the first surgery and positive to stimulation at the second surgery (Site 1); loss of function sites were the inverse (Site 2). Cross-modal sites were positive to stimulation at both surgeries, but function changed from motor to sensory function or vice versa (Site 3).

Digital grids composed of 1 × 1 cm regions of interest were created and superimposed on the digital photographs of the intraoperative mapping results (example, Figure 1). Grids were placed parallel to the central sulcus, allowing inclusion of the pre- and postcentral gyri. (More details of stimulation electrodes in this paragraph.) An identical grid orientation was utilized during analyses of stimulation sites from both operations. A positive site was noted if the respective 1 × 1 cm box contained a positive stimulation site (sensory or motor). Negative sites contained no sensory or motor response to stimulation. Two independent researchers performed the analysis, and discrepancies were resolved with a third researcher to reduce bias. Blinding or randomization was not necessary in this retrospective study. These grid stimulation points were then classified into five primary categories: gain of function, loss of function, change of function, same function, and lack of function. Gain of function sites were negative at the first operation and positive at the second. Loss of function sites were positive at the first operation and negative at the second. Change of function sites were positive at both operations; however, the specific function changed from sensory to motor or motor to sensory. Same function sites were positive at both operations and maintained either motor or sensory function. Lack of function sites were negative at both operations. Loss of function and lack of function sites were further classified into those with or without a corresponding motor or sensory deficit on clinical neurologic exam.

### 2.4. Tumor Volume Calculation

The volume of the lesion was determined using Brainlab Elements software. Postcontrast axial T1 3-D MPRAGE MRI images were analyzed. Outlines of the tumor were drawn on consecutive axial images through the tumor and a volume calculated using standard clinical neuroimaging software (Brainlab, Munich, Germany—License provided by Northwestern Memorial Hospital).

### 2.5. Plasticity Definition and Modalities

We define plasticity as a gain, loss, or change of function of the same stimulation site between the two surgeries without a corresponding neurologic change (Figure 1). In other words, plasticity is a functional change at the cortical level, as determined by stimulation, while motor and/or sensory function was maintained on clinical neurologic exam. Additionally, sites that lacked function at both surgeries without a corresponding clinical neurologic deficit were considered as having already exhibited plasticity at the first operation. For example, if the hand knob was stimulated at both surgeries and did not elicit hand movement, but the patient still had full hand motor function on neurologic exam in clinic, this was considered plasticity. Thus, in this example, hand function was reorganized to another cortical region.

**Figure 2:**
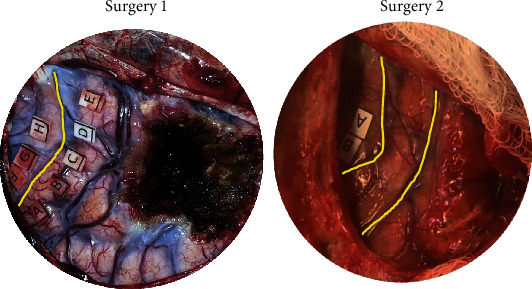
Plasticity, distant recruitment. The yellow box highlights the same portion of the left postcentral gyrus. This figure demonstrates distant recruitment plasticity of Patient 1. At surgery 1, there was complete activation of the exposed postcentral gyrus (Points A-E). At surgery 2, stimulation could no longer elicit function in the identical region of the postcentral gyrus. However, Patient 1 had no sensory deficits at surgery 2. Thus, the function that had been in the postcentral gyrus at surgery 1 had reorganized distally (outside of the operative field) by surgery 2 to preserve sensory function. This is a prototype of distant recruitment plasticity.

**Figure 3:**
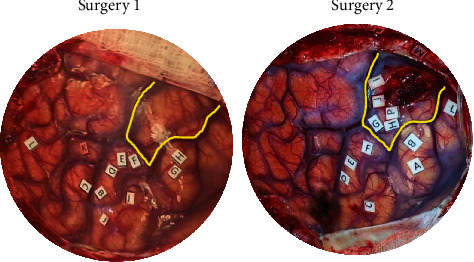
Plasticity, near recruitment. The yellow box highlights the same cortical area overlying the tumor. At the first surgery, there is no functional activation in this area. At the second surgery, both motor and sensory functions are recruited into this perilesional cortical area (Points G-J, P). This is a prototype of near recruitment plasticity.

Two broad plastic mechanisms were assigned from these data—either distant or near recruitment of function. Distant plastic recruitment was assigned if a patient exhibited plasticity but the new functional sites were not in the exposed region of cortex; this plasticity was said to have been recruited from a distant, nonexposed cortical region (Figure 2). Patients who had new positive sites within the exposed area at the second surgery were considered as having near recruitment of function (Figure 3).

## 3. Results

### 3.1. Patient Demographics

Due to strict inclusion criteria, six patients were both eligible and included in this study from 2010 to 2016. Five patients were male and one was female. The average tumor volume was 21.8 ± 18.6 cm^3^. All patients had tumors that were histology-proven GBM at both operations. The tumors were distributed throughout the frontal, temporal, and parietal lobes; four were located in the left hemisphere and two were in the right hemisphere. Five patients were right-handed, and one was left-handed. The average age at the time of the first surgery was 46.7 ± 15.2 years. The average interval between the first and second surgeries was 219.8 ± 37.5 days. Patients 4, 5, and 6 had a previous craniotomy for tumor resection by a different surgeon prior to their repeat awake craniotomies in this study (Table 1). Between the two awake mapping surgeries, only one patient (Patient 5) completed a stay at acute inpatient rehabilitation; the remaining five patients in the study were discharged to home. All patients received medical therapy between mapping sessions, and half of the patients received concurrent fractionated radiation therapy.

### 3.2. Stimulation Points Summary

A total of 51 positive stimulation sites were recorded across both surgeries for all 6 patients. The average number of sites stimulated per patient was 8.8 ± 6.4 sites, with a range of 5-19 sites across patients. Of 51 sites, 32 (62.7%) were plastic and 19 (37.3%) were nonplastic. Of 51 sites, 21 (41.2%) were in the primary sensory cortex and 30 (58.8%) were in the primary motor cortex. For the plastic sites, 18 (35.3%) were in the sensory cortex and 14 (27.5%) were in the motor cortex. For the nonplastic sites, 3 (5.9%) were in the sensory cortex and 16 (31.3%) were in the motor cortex.

A site was considered plastic if it gained function, lost function with no corresponding neurologic deficit observed in the patient, or changed function. A total of 9 sites (17.6%) gained function; 4 sites were in the sensory cortex and 5 were in the motor cortex. A total of 19 sites (37.3%) lost function at either the first or second surgery, but importantly had no corresponding clinical neurologic deficit. Of these 19 sites, 12 were sensory and 4 were motor. A total of 4 sites (7.8%) changed function; 2 sites elicited a different motor function, and 2 others changed from motor to sensory function.

A site was considered not plastic if it had the same function or if it lost function and there was a corresponding functional deficit. In total, 14 sites (27.5%) lost function concomitant with a functional deficit observed in the patient; all were in the motor cortex. Also, 5 sites (9.8%) had the same function at both surgeries; three were in the sensory cortex and two were in the motor cortex (Table 2).

### 3.3. Mechanisms and Sensorimotor Representation of Plasticity

Of the 6 patients, 5 patients demonstrated plasticity. By definition, the patients who demonstrated plasticity had no deficits in motor or sensory function on their clinical neurologic exam. Two primary mechanisms of plasticity, distant and near recruitment of function, were observed. Distant recruitment was the most common mechanism of plasticity, representing 4 of 5 (80%) patients. One patient (20%) had near recruitment of function. An example of distant and near recruitment from our patient cohort is provided in Figures 2 and 3, respectively. One patient also demonstrated cross-modal plasticity, with 2 sites switching from motor to sensory function.

Plasticity occurred both in the primary motor and primary somatosensory cortices. All patients who demonstrated plasticity had plastic reorganization both in the motor and sensory cortices. Furthermore, one patient had two sites of cross-modal plasticity, in which motor sites became sensory. Interestingly, the only patient who exhibited cross-modal plasticity was also the only one who demonstrated near recruitment of function. Overall, the plasticity demonstrated by 5 of the 6 patients was robust (90, 75, 60, 50, and 36% of total sites) (Table 3).

## 4. Discussion

We demonstrate that plasticity within the adult human primary motor and somatosensory cortices is not only possible but in fact relatively common. This is the first study to our knowledge that investigates M1 and S1 plasticity through DES in GBM patients. The understanding that neuroplasticity can occur in GBM, especially within the primary cortex, is unprecedented and will lead to further understanding of how the adult brain can adapt to this common and aggressive primary brain tumor.

Specifically, the key findings of our study are twofold: (1) plasticity can indeed occur in GBM, a rapidly growing and aggressive tumor, and (2) plasticity can occur in the primary motor and sensory cortices in the setting of GBM and is in fact a relatively common mechanism to preserve function in this cohort. This is the first study to utilize the most direct measure of human cortical function, DES, to examine neuroplasticity in the most common primary brain tumor, GBM. Prior studies used DES to demonstrate motor and somatosensory cortex plasticity in glioma, but these data were in low-grade glioma patients [12, 15, 16, 17]. In terms of GBM, only one study to date has shown primary motor cortex plasticity in GBM. This was a study which included a single GBM patient and used an indirect measure of cortical activity—functional MRI [21]. To our knowledge, there are no studies to date that have reported primary sensory plasticity in GBM. Thus, our data of both primary motor and sensory plasticity in GBM is novel and adds to the growing literature on plasticity in the setting of infiltrating gliomas.

In addition to the presence or absence of plasticity, one question that arises with regard to neural plasticity in brain tumor patients is the time scale of changes. An excellent and relevant study in 2016 demonstrated plasticity in grades 2 and 3 gliomas via repeat DES with a mean interval of 4.1 years [12]. More recently, a transcranial magnetic stimulation study showed plasticity in LGG patients with an average mapping interval of over two years [22]. TMS has also shown cortical reorganization in HGG over a large range of time (3-42 months) [23]. Our data using DES—the gold standard for cortical mapping—supports these findings. We further demonstrate that plasticity occurs commonly in HGG and that these changes occur relatively quickly, over months. The average intraoperative interval was only nine months for our patients who developed plasticity. For patients that already had plasticity at the first operation, this plasticity may have occurred concurrently with the growth of an aggressive malignancy. High-grade gliomas grow quickly—even small, untreated tumors can double in size in 50 days—thus, the brain would have to quickly adapt to reorganize function in coordination with expanding and infiltrating tumor [24].

It is important to address that our definition of plasticity is inherently very conservative. Any motor or sensory deficit would disqualify a patient from having any type of motor or sensory plasticity. The rationale for this conservative definition was to avoid classification error and to ensure we were identifying pure motor and sensory plasticity. In doing this, we likely underestimated the frequency of plasticity because we may have disqualified patients who had a deficit in addition to plasticity.

Briefly, it is important to mention potential mechanisms of neuroplasticity from both the anatomic to the cellular levels. Our work, as described above, characterizes both near and distant recruitment of function. This anatomic model has been hypothesized to be achieved by both recruitment of nearby cortical circuits and uncovering of redundant cortical circuits. Cellular mechanisms of plasticity rely on the concept of long-term potentiation, through which synaptic receptors are up- or downregulated based on neuronal input. Specifically, the AMPA and NMDA glutamate receptors play key roles in LTP [25, 26]. It will be important in the future to dissect the specific mechanisms contributed to our observed effects.

The most important finding of this work is that the primary motor and somatosensory cortices are capable of reorganizing in response to GBM. This is the first study to examine M1 and S1 neuroplasticity in GBM patients using DES across two awake craniotomies. Not only is plasticity present, it is robust; 62.7% of sites exhibited. It also occurs relatively quickly; the average time between operations was 219.8 days—just over seven months.

The future potential clinical applications of these data are very interesting. Operations for GBM straddle the line between aggressively resecting tumor and preserving function. Gross-total resection is the most significant surgical factor that can improve patient survival in GBM [2, 27]. However, subtotal resections are often the only option since delicate care is taken in primary cortical areas, like M1 and S1, as to not injure their crucial function. The plasticity seen in our study suggests that M1 and S1 can redistribute function beyond what was previously thought possible. Thus, plasticity of M1 and S1 could be taken advantage of to allow for more aggressive resections in GBM located near the primary somatosensory cortices and/or to better plan surgical interventions in the recurrent setting. Additional studies of the mechanisms underlying sensorimotor plasticity in glioma patients may lead to strategies that actively promote local or distant reorganization of sensorimotor function, whether preoperatively to increase extent of resection or postoperatively in the rehabilitation setting, to improve functional outcomes in GBM patients.

## Figures and Tables

**Table 1:** Patient demographics. Six patients fit the inclusion criteria for this study between 2010 and 2016. Listed are their sex, age at first operation (years), operative interval (days between the first and second operations), tumor pathology, tumor volume (cm^2^), and tumor location (if tumor was not specifically in the pre- or postcentral gyrus, lobe is listed).

Patient #	Sex	Age, first surgery	Op interval (days)	Tumor pathology	Previous surgery?	Tumor volume (cm^3^)	Tumor location
1	M	54	278	GBM	No	35.9	L frontal
2	M	55	198	GBM	No	51.9	R postcentral
3	M	63	231	GBM	No	18.2	L precentral
4	M	21	170	GBM	Yes	13	R parietal
5	F	50	204	GBM	Yes	2.31	L postcentral
6	M	37	238	GBM	Yes	9.38	L temporal

**Table 2:** Stimulation points' summary. At the time of the second operation, sites were classified as plastic if they gained function, lost cortical function without clinical deficit, or changed function. Nonplastic sites were those that lost cortical function with a clinical deficit or had the same function at the second operation.

Interoperative change	Plasticity			No plasticity	
	Gain of function	Loss of function	Change of function	Loss of function	Same function
Sensory, # of sites	4	12	2	0	3
Motor, # of sites	5	7	2	14	2

**Table 3:** Mechanisms and sensorimotor representation of plasticity. We categorized the stimulation sites into plastic or nonplastic. Overall, 63% of sites were plastic, with motor and sensory plasticity distributed as shown. The cross-modal plasticity seen was a motor-to-sensory change. Based on the observed mechanism of plasticity, either distant or near recruitment was assigned.

Patient #	Plasticity % (of total sites)	Motor sites (of plastic)	Sensory sites (of plastic)	Cross-modal sites (of plastic)	Recruitment
1	50%	50%	50%	0%	Distant
2	36%	25%	75%	0%	Distant
3	74%	22%	64%	14%	Near
4	60%	67%	33%	0%	Distant
5	0%	0%	0%	0%	None
6	90%	67%	33%	0%	Distant

## Data Availability

The data that support the findings of this study are available from the corresponding author, upon reasonable request.

## Abbreviations

DES: Direct electrocortical stimulation
GBM: Glioblastoma multiforme
HGG: High-grade glioma
LGG: Low-grade glioma.
